# Highly Stable Pickering Emulsions with Xylan Hydrate Nanocrystals

**DOI:** 10.3390/nano11102558

**Published:** 2021-09-29

**Authors:** Shanyong Wang, Zhouyang Xiang

**Affiliations:** State Key Laboratory of Pulp and Paper Engineering, South China University of Technology, Guangzhou 510640, China; feshanyong@mail.scut.edu.cn

**Keywords:** hemicellulose, xylan, nanocrystal, Pickering emulsion

## Abstract

Xylan is a highly abundant plant-based biopolymer. Original xylans in plants are in an amorphous state, but deacetylated and low-branched xylan can form a crystalline structure with water molecules. The utilizations of xylan have been limited to bulk applications either with inconsistency and uncertainty or with extensive chemical derivatization due to the insufficient studies on its crystallization. The applications of xylan could be greatly broadened in advanced green materials if xylan crystals are effectively utilized. In this paper, we show a completely green production of nano-sized xylan crystals and propose their application in forming Pickering emulsions. The branches of xylan were regulated during the separation step to controllably induce the formation of xylan hydrate crystals. Xylan hydrate nanocrystals (XNCs) with a uniform size were successfully produced solely by a mild ultrasonic treatment. XNCs can be adsorbed onto oil–water interfaces at a high density to form highly stable Pickering emulsions. The emulsifying properties of XNCs were comparable to some synthetic emulsifiers and better than some other common biopolymer nanocrystals, demonstrating that XNCs have great potential in industrial emulsification.

## 1. Introduction

Hemicelluloses are a type of very abundant plant-based biopolymers [1,2]. Xylan is a major type of hemicellulose and is primarily found in hardwoods and graminaceous plants. Xylan is also present in softwoods but in relatively low amounts. Depending on the plant species, xylans have *β*-(1-4)-linked D-xylopyranosyl backbones but are substituted with one or more monomers of L-arabinofuranosyl, D-glucuronopyranosyl, (4-O-methyl)-D-glucuronopyranosyl, and some minor residues [3]. The backbones of xylans in hardwood and graminaceous plants are partially acetylated, but softwood xylans are not acetylated. Original xylans in plants are in an amorphous state [4], but deacetylated and low-branched xylans can form a crystalline structure with solvent molecules, such as water and dimethyl sulfoxide (DMSO) [5,6,7]. Due to the insufficient studies on xylan crystallization, the applications of xylans have been mainly focused on bulk applications, such as films and coatings [8,9]. However, xylan crystallization affects its solubility and viscosity [10] and is detrimental to its water cast film formation [6], adding inconsistency and uncertainty to its bulk applications. The applications of xylan could be greatly broadened if xylan crystals are nano-sized, as nanocrystals of some biopolymers, e.g., cellulose, chitin/chitosan, and starch, have been widely used in advanced functional materials, such as biological scaffolds, drug carriers, optical materials, permselective membranes, polymer electrolytes, and emulsion stabilizers. [11,12]. However, preparing xylan nano-crystals with controllable size and shape is difficult and has only been reported on in a few studies [7,13]. Chanzy et al. reported the desizes of xylan crystals by enzymatic degradation, but the enzyme degrades the xylan crystals from the edge and destroys the crystalized structure [13]. Wang et al. reported the nano-sizing of xylan hydrate crystals by surface carboxymethylation, but this method resulted in a significant decrease in crystallinity and uncontrollable sizes [14]. Meng et al. recently found that well-dispersed alkali-extracted xylan in DMSO can spontaneously form xylan–DMSO crystal nanowhiskers with a controllable size by heating induction [7]; the mechanisms behind it were still not fully understood and the application of the xylan–DMSO crystal in an aqueous environment is not known. The limited knowledge of the controllable preparation of nano-sized xylan crystals has largely impeded its applications in advanced materials.

Many of the bio-based nanocrystals, e.g., cellulose nanocrystals (CNC) [15] and chitin nanocrystals [16], require severe acid treatments or chemical derivatizations to produce, causing chemical recycling and pollution issues. Due to a much less bulky structure of xylan, compared to cellulose and chitin, we hypothesized that the nano-sizing of xylan hydrate crystals to produce xylan hydrate nanocrystals (XNCs) only requires a mild physical treatment with a low energy input, which would be a completely green process. However, xylan is not always in a crystalized form [6], so controllably separating xylans with a low degree of branching to induce crystallization is an important prerequisite to the production of XNCs.

CNCs [15,17,18], chitin nanocrystals [16,19], and starch nanocrystals [20,21] have already been reported to be effective in forming and stabilizing Pickering emulsions. During the emulsifying process, the nanocrystals were adsorbed onto the oil–water interface, irreversibly, and formed a highly rigid layer to prevent the aggregation between discontinuous phases [12]. However, the application of XNCs in the Pickering emulsion has never been reported. Since the crystallization of xylans requires the involvement of solvent molecules, they have a high stability in the corresponding solvents [5,7,22]. Xylan hydrate crystals are very stable in water, as demonstrated by their water insolubility [6,10], so their nano-sized form is hypothesized to be especially suitable in the forming of oil–water Pickering emulsions.

## 2. Experimental Section

### 2.1. Material

Glacial acetic acid (AR), sodium chlorite (80%), sodium hydroxide (AR), sodium dodecyl sulfate (SDS, AR), Tween 80 (T80, pharmaceutical grade), Triton X-100 (TX100, AR), gum arabic (GA, pharmaceutical grade), fluorescent brightener VBL, styrene, and 2,2′-Azobis (2-methylpropionitrile) (ABIN, AR) were purchased from Shanghai Macklin Biochemical Co., Ltd. (Shanghai, China) Medium-chain triglycerides (the carbon number of each fatty acid chain is 8–10) were purchased from Shanghai Yuanye Bio-Technology Co., Ltd. (Shanghai, China) Absolute ethanol (AR) and hydrochloric acid were of analytical grade.

### 2.2. Separation and Characterization of Xylan

A grounded sugarcane bagasse sample, sieved between 40 and 60 mesh, was extracted in a Soxhlet extractor with 95% ethanol for 6 h to remove extractives, fat, and wax [6]. Holocelluloses were separated by treating the bagasse in a 2 wt% sodium chlorite solution with pH = 3.9–4 and a solid–liquid ratio of 1:20 at 75 °C for 1 h. The holocelluloses were washed three times with deionized water and one time with absolute ethanol and then air dried.

Xylans were separated from holocelluloses by alkaline extraction or successive alkaline extraction with NaOH solutions of different concentrations at 90 °C for 3 h (Table 1) [6,23]. After the extraction, the filtrate was adjusted to pH 5.5–6 by HCl and then concentrated to one-third of the original volume. The filtrate was then added to absolute ethanol with volumes three times of the filtrate and then stood overnight. The precipitate was centrifuged (Centrisart G-16, Sartorius, Gottingen, Germany) at 4200 rpm for 10 min, washed with 70% ethanol (*v*:*v*) three times, freeze dried, and ground to powders. The white powders obtained were xylans.

The xylans were hydrolyzed according to the protocols described previously [24] in order to determine their lignin, neutral sugar, and uronic acid compositions. Neutral sugars were quantified by an ion chromatography system (IC-3000, Dionex, Sunnyvale, CA, USA) equipped with an anion-exchange column (CarboPacTM PA20, Dionex, Sunnyvale, CA, USA); the column was pre-equilibrated with 200 mM NaOH and gradiently eluted with water, 20 mM NaOH, and 500 mM sodium acetate at a flow rate of 0.5 mL/min. The estimation of the uronic acid content was based on the photometric method developed by Filisetti-Cozzi [25]. The molecular weight distribution of xylans was measured by GPC (PL-GPC50, Agilent Technologies, Santa Clara, CA, USA), with DMSO as the mobile phase.

The morphology of xylan particles in suspension was observed with a polarized microscope (BX53M, Olympus, Tokyo, Japan) equipped with a U-AN360P analyzer slider. X-ray diffraction (XRD) measurements on xylan powders were performed in the symmetric reflection mode using X’Pert Powder (Panalytical, Eindhoven, The Netherlands) equipped with a copper X-ray source (k = 1.5418 Å) and operated at 40 kV and 40 mA.

### 2.3. Preparation and Characterization of XNCs

The xylan aqueous suspensions were sonicated at 15 W/mL by an ultrasonic cell grinder (JY99-IIDN, Ningbo Xinzhi Biological Co., Ltd., Ningbo, China) for 40 min to obtain a stable XNC colloidal dispersion. The XNC colloidal dispersion was diluted 500 times and then dropped and air dried on a mica sheet, which was analyzed by an atomic force microscope (AFM, Nanoscope IIIa, Bruck, Germany). The particle size distribution of XNCs was calculated from the AFM images using Nanoscope Analysis 1.7. The diluted XNC colloidal dispersion was stained with phosphotungstic acid and then analyzed by transmission electron microscopy (TEM). The viscosity of the XNC dispersion was measured by a digital rotational viscometer (LV-SSR, Shanghai Fangrui Instrument Co., Ltd., Shanghai, China).

### 2.4. Preparation and Characterization of Pickering Emulsion

Emulsions were prepared with triglycerides at an oil–water ratio of 2:8 under different emulsifier concentrations (0.5, 1, 2 and 4 wt%) and was homogenized by an ultrasonic cell grinder at 10 W/mL for 90 s. The emulsifying activity (EA), emulsion cream index (ECI), and emulsion droplet size were evaluated. The EA was measured according to the method of Hu [26]. Approximately 30 μL of emulsion was mixed with 15 mL in a 0.1 wt% SDS aqueous solution, and the absorbance was measured at 500 nm by a UV–VIS spectrophotometer (Shanghai Mapada Instrument Co., Ltd., Shanghai, China). The ECI was calculated based on the previous methods [17,18,26] with slight modifications. The prepared emulsion was continuously centrifuged at 4000 rpm, the layering of the emulsion at different centrifugation times was recorded, and the *ECI* was calculated as follows:

$$ECI=\frac{H_{A}}{H_{T}}\times 100\%$$

where *H_A_* is the height of the upper phase, and *H_T_* is the total height of emulsion. The emulsion droplet size was measured by a laser particle size analyzer (LA960S, HORIBA, Kyoto, Japan) at a refractive index of 1.5 and a solid content of 0.5 wt%.

The aqueous solution of fluorescent brightener (1 mg/mL) was used to dye the XNC colloidal dispersions (the volume ratio of brightener solution to dispersion was 1:100). The dyed XNC colloidal dispersion was used to prepare the Pickering emulsions. The fluorescence distribution of XNCs in the emulsion was observed by a laser confocal microscope (TCS SP5, Leica, Munich, Germany) at an excitation wavelength of 403 nm and a collection wavelength of 420–500 nm. According to previous conditions [17], the oil phase of the emulsion was replaced by styrene that was initiated with polymerization. A field-emission scanning electron microscope (FESEM, Merlin, Zeiss, Oberkohen, Germany) was used to observe the adsorption of XNCs on the surface of polystyrene droplets. In order to observe the emulsion droplet surface more easily, the dyed emulsions and the polymerized emulsions were dispersed by a lab high-speed homogenizer (T18, IKA, Guangzhou, China) instead of using ultrasonic treatment to obtain larger oil droplets.

## 3. Results and Discussion

### 3.1. Xylan Crystallinity

**Figure 1:**
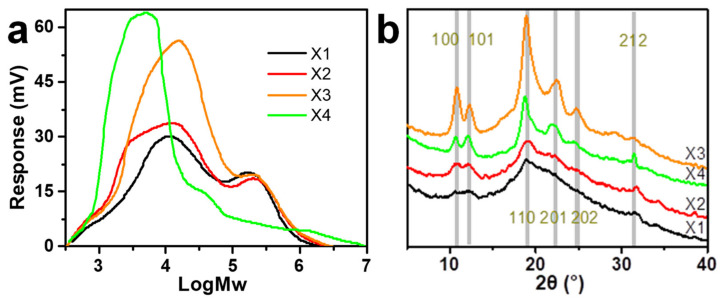
(**a**) Molecular weight distribution and (**b**) XRD profiles of different xylan samples.

The chemical structures of xylans were regulated by alkaline extraction or successive alkaline extraction with NaOH solutions of different concentrations, giving xylan samples X1–X4 (Table 1). A high concentration of NaOH solution extracted xylans with less branches and a relatively lower molecular weight (X3 and X4), compared to those extracted by a low concentration of NaOH solution (Table 2 and Table 3; Figure 1a), which was consistent with previous studies [6].

The XRD profiles of the four xylan samples are shown in Figure 1b. X1 and X2 had profiles showing mostly amorphous states, while X3 and X4 had peaks characterizing typical xylan hydrate crystals [6]. The Miller indices of major peaks based on the trigonal unit cell of the xylan hydrate crystal in the study of Nieduszynski (1972) [5] are indicated. When observed under crossed polarizers, the suspension of X1 and X2 showed little birefringence, while X3 and X4 showed a large number of birefringent granular objects (Figure S1), further suggesting the high crystallinity of X3 and X4 samples. The crystallinity of xylan was positively correlated with the degree of branching, which is consistent with previous studies [6,27]. The crystallinity of X4 was slightly lower than that of X3, which may be related to the degradation of the X4 molecular chain (Table 2 and Figure 1a). Previous studies have shown that reducing the length of the polymer chains would increase their degree of freedom and, subsequently, reduce their possibility of aggregating into ordered structures [28], which indicates that the low molecular weight might not be conducive to the crystallization of xylan. These results indicated that regulating the degree of branching to induce the formation of xylan hydrate crystals was successful, facilitating the subsequent nano-sizing of xylan hydrate crystals.

### 3.2. XNC Preparation

**Figure 2:**
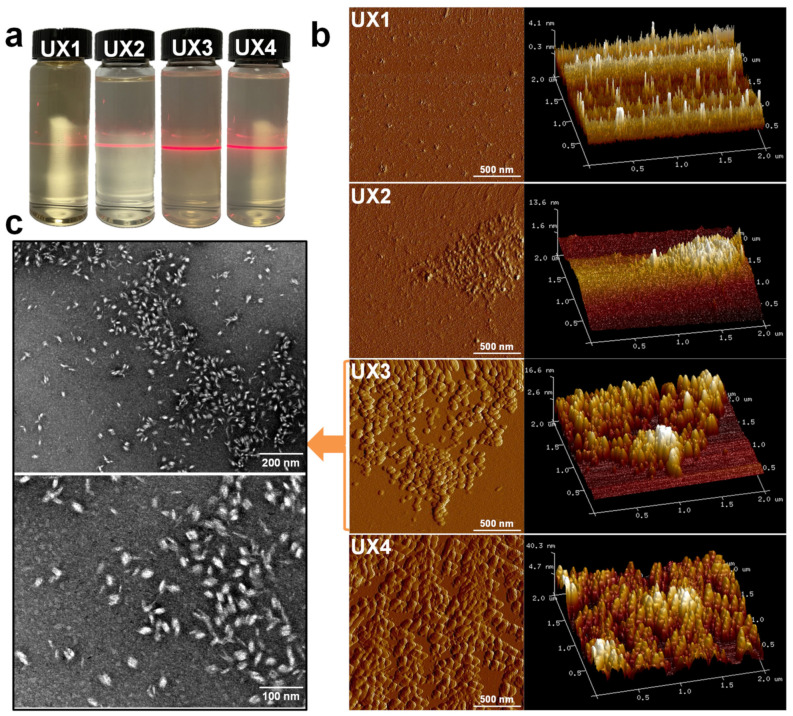
(**a**) Tyndall phenomenon, (**b**) AFM images, and (**c**) TEM images of xylan colloidal dispersions.

**Figure 3:**
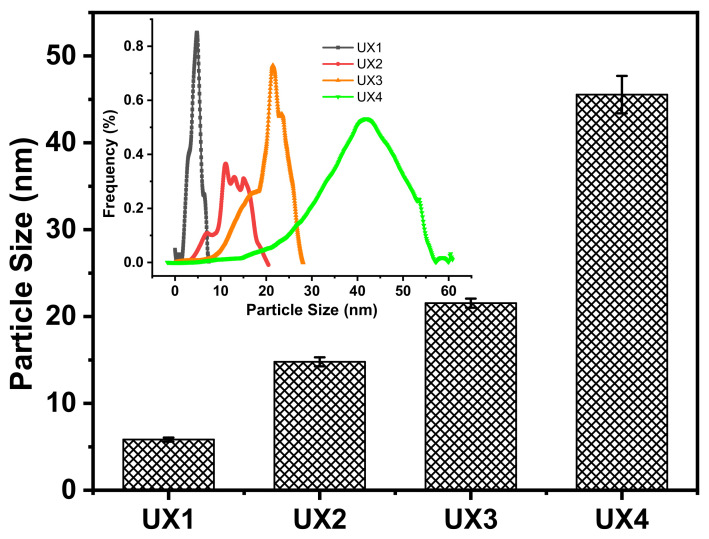
Particle size distribution and the average particle size of xylan colloidal dispersions.

Ultrasonic waves were introduced to the suspensions of xylan crystals in order to nano-size the xylan hydrate crystals to xylan hydrate nanocrystals (XNCs). The power of the ultrasonic treatment was carefully controlled at a mild level, because a higher power would have destroyed the crystals and a lower power would not have been able to nano-size the crystals. Before the ultrasonic treatment, xylans X1 and X2 were dissolved in water into a transparent solution upon heating, while X3 and X4 could not form stable suspensions but deposited at the bottom of water (Figure S2), again suggesting that xylan solubility relates to its crystallinity (Figure 1b). After ultrasonic treatment, the evident optical path of the red laser through the treated suspensions indicated the formation of stable colloidal dispersions UX1-4 (Figure 2a). AFM and TEM images of UX3 and UX4 demonstrated nanoparticles with high uniformity and a spindle shape, indicating that uniformly sized XNCs had been successfully prepared (Figure 2b,c). UX4 showed a particle diameter (~45.5 nm) larger than that of UX3 (~21.5 nm); UX4 also showed a wider particle size distribution than that of UX3 (Figure 3). UX1 and UX2 both showed a few shapeless particles in the field of vision, a very narrow particle size distribution, and a small particle size, suggesting the dissolution or degradation of noncrystalline xylan particles (Figure 2b and Figure 3). The diameter of XNCs showed a positive correlation to the crystallinity of starting xylan samples (Figure 1b).

In sum, there are mostly noncrystalline and soluble xylans in UX1 and UX2 colloidal dispersions, while there are mostly XNC in UX3 and UX4 colloidal dispersions. The XNC is a bio-based nano-material similar to the cellulose nanocrystal (CNC). Different from cellulose, xylan is not always in a crystalized form [6], so controllably separating xylans with a low degree of branching to induce crystallization is an important prerequisite to the production of XNCs. However, the production of CNCs requires a concentrated acid treatment. Due to the less bulky structure of xylan, compared to cellulose, XNC production only requires ultrasonic treatment with a mild energy input, which is completely green without any acid treatments and chemical derivatizations.

### 3.3. Emulsifying Properties of XNCs

**Figure 4:**
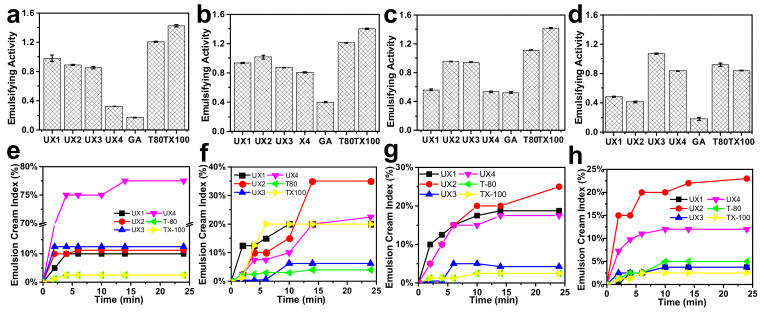
Emulsifying activity (**a**–**d**) and emulsion cream index after centrifugation (**e**–**h**) at 0.5, 1, 2, and 4 wt% emulsifier concentrations.

**Figure 5:**
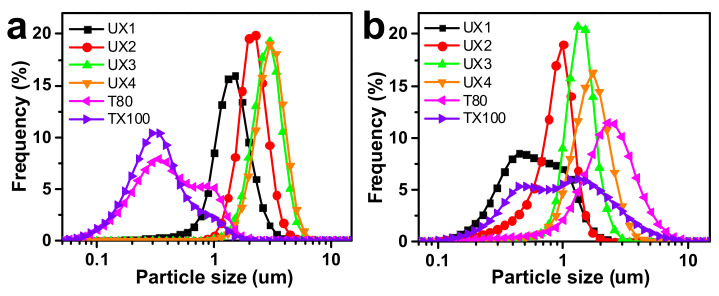
Particle size distribution of emulsions with (**a**) 0.5 wt% and (**b**) 2 wt% emulsifiers.

The xylan colloidal dispersions (UX1–UX4) were used as an emulsifying agent to produce oil-–water emulsions and were compared with gum arabic (GA), Tween 80 (T80), and Triton X-100 (TX100). GA is a type of polysaccharide collected from *Acacia senegal* and is one of the most common natural food emulsifiers. T80 and TX100 are typical synthetic non-ionic surfactants. The obtained emulsions were tested for emulsifying activity (EA) and emulsion cream index (ECI), where EA refers to the ability of the emulsifier to form an emulsion and ECI corresponds to the stability of the formed emulsion [29]. For EA (Figure 4a–d), xylans had much better EA than GA at all emulsifier concentration levels. Xylan dispersions showed lower EA values than synthetic emulsifiers at low emulsifier concentrations, but UX3 showed the highest EA (1.07) and UX4 showed a comparable EA (0.84) to synthetic emulsifiers (0.92 for T80 and 0.84 for TX100) at a high emulsifier concentration of 4 wt%. For ECI (Figure 4e–h), in general, xylan dispersions showed emulsifying stabilities comparable to the synthetic emulsifiers, except at a very low emulsifier concentration of 0.5 wt%. UX3 in particular showed an excellent ECI that was maintained below 5% after centrifugation for 24 min. UX1 also showed an ECI maintained below 5% after 24 min, but only at a high emulsifier concentration of 4 wt%. The emulsifying stability of xylan dispersions can also be proved by the pictures of emulsions after standing for seven days (Figures S3 and S4). The particle size of the emulsion is also an important indicator for the evaluation of the formed emulsions [29]. Xylan-assisted emulsions showed larger and comparable particle sizes at emulsifier concentrations of 0.5% and 2%, respectively, compared to those of T80 and TX100, but they also showed narrower particle distributions than those of T80 and TX100 (Figure 5).

**Figure 6:**
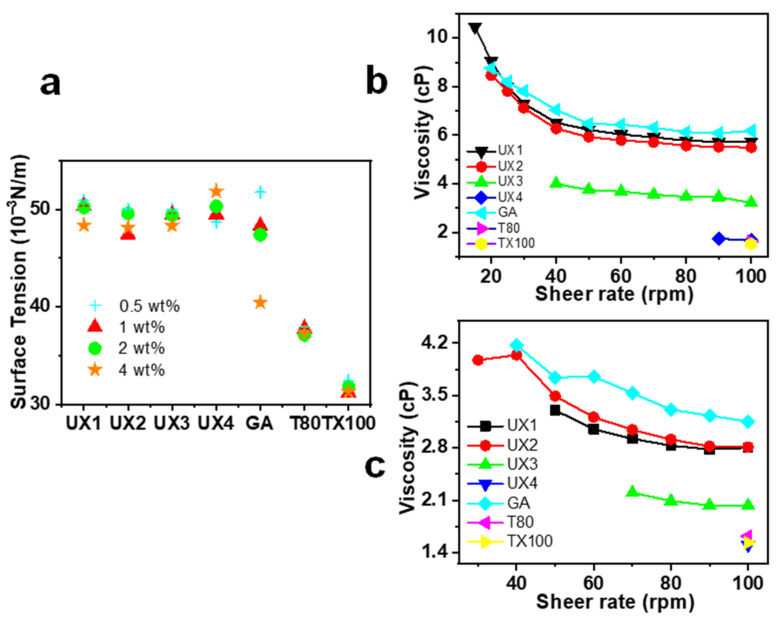
Surface tension (**a**), rheological viscosity of xylan colloidal dispersions, and other emulsifiers at 4 wt% (**b**) and 2 wt% (**c**) concentrations.

In general, xylan colloidal dispersions had much better emulsifying properties than those of GA and had comparable emulsifying properties to synthetic emulsifiers (Figure 4). T-80 and TX100 have a high surface activity as shown by their low surface tension (Figure 6a) and, therefore, can be quickly adsorbed onto the oil–water interface to assist the stabilization of an emulsion [30]. The emulsifying properties of xylan dispersions are quite dependent on their concentrations, which may be due to the fact that the viscosity of xylan dispersions is concentration dependent and shear thinning (Figure 6b,c). Previous research has shown that the rheological property of emulsifiers plays an important role in stabilizing emulsions, besides interfacial adsorption [31,32]. This suggests that UX1 has a good ECI, which is comparable to that of T80 and TX100 only at a high emulsifier concentration. This also explains the better emulsifying properties of UX3 than UX4, since UX3 has a higher viscosity than that of UX4 (Figure 4b–d and Figure 6b,c).

**Figure 7:**
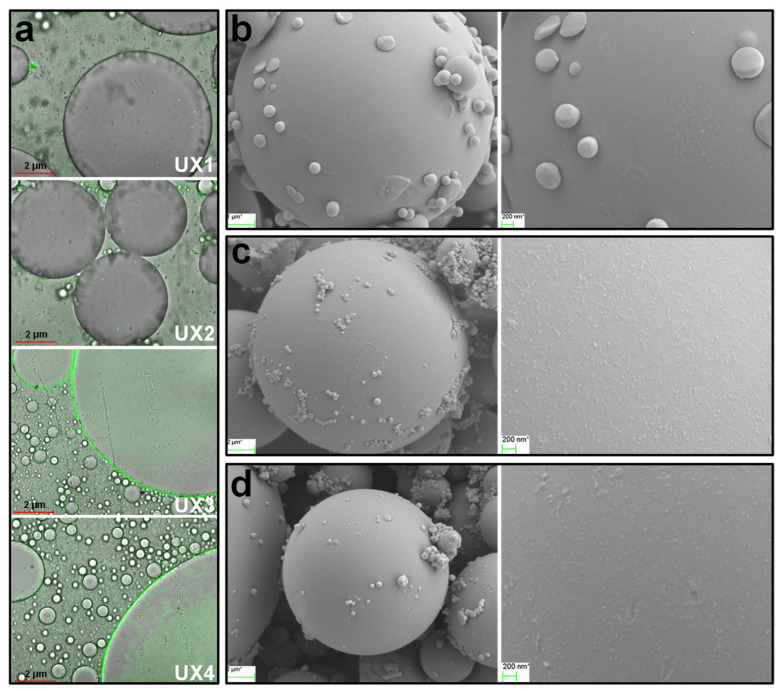
(**a**) Laser confocal microscope images of UX1–UX4 adsorption on the oil droplet surface. FESEM images of the rigid polystyrene droplet (produced from in situ polymerization) surface: (**b**) no emulsifier, (**c**) UX3, and (**d**) UX4 as the emulsifier.

Comparing the different xylan colloidal dispersions, UX3 and UX4 had better emulsifying properties than UX1 and UX2, which may be due to the formation of uniformly sized XNC particles in UX3 and UX4 (Figure 2). The assistance of XNC particles in the emulsions can be better proved by dyeing the emulsifiers beforehand and observing their distribution in the emulsion by fluorescence [15]. As shown in Figure 7a, no obvious fluorescence was observed on the oil droplet surface of the UX1 and UX2 emulsions. On the contrary, an obvious green fluorescence appeared on the oil droplet surface with full coverage for the UX3 and UX4 emulsions. This suggested the forming of Pickering emulsions with the assistance of XNCs.

To further analyze the assistance of XNCs in Pickering emulsions, the oil phase was replaced with styrene, which was emulsified with water by UX3 and UX4. Styrene was polymerized in situ within its droplets, and rigid polystyrene microspheres were obtained (Figure 7b). The polystyrene microspheres showed a large size due to the avoidance of severe mechanical treatment during polymerization in the formed emulsion, which was conducive to observing the distribution of XNCs onto the surface of the microspheres [17,33]. By adding XNCs as the emulsifier, XNC particles adsorbed onto the surface of the polystyrene microspheres were clearly observed (Figure 7c,d). The dense coverage of XNCs on the oil (styrene) droplet surface formed a strong barrier layer to keep the styrene droplets in a spherical shape during the process of styrene polymerization. This again proves the high-density adsorption of XNCs on oil-–water interface, preventing the aggregation of oil droplets and the forming of stable Pickering emulsions assisted by XNCs. In addition, UX3 emulsion droplets adsorbed more XNC particles on the surface than UX4 emulsion droplets (Figure 7c,d), which indicates that an XNC with a smaller size is more conducive to its adsorption onto the oil-–water interface. This is another explanation of the better emulsifying properties of UX3 in comparison to UX4.

Noncrystalline and soluble xylans are able to provide emulsification [32], but our study shows that, without the ability of forming Pickering emulsions, noncrystalline xylans, i.e., UX1 and UX2, demonstrate lower emulsifying properties than XNCs, i.e., UX3 and UX4. As shown in Figure 7a, UX1 and UX2 as emulsifiers have almost no oil-–water interfacial adsorption in the emulsions. That is, the stability of UX1 and UX2 emulsions depends mainly on the gel network formed by the emulsifier, namely viscosity [31,32]. For UX3 and UX4 emulsions, with the assistance of XNC interfacial adsorption, their emulsion stability is greatly improved.

In comparison with some other common biopolymer nanocrystals, e.g., CNCs, chitin nanocrystals, and starch nanocrystals, UX3 and UX4 had better emulsifying properties (Table 4). In comparison with synthetic emulsifiers, e.g., T80 and TX100, UX3 and UX4 demonstrated comparable emulsifying properties. Considering that the production of XNCs is completely green without any acid treatments or chemical derivatizations, we state that XNCs have great potential to substitute synthetic chemicals in emulsification.

## 4. Conclusions

The formation of xylan hydrate crystals can be induced by regulating the degree of branching of xylans. Xylan hydrate nanocrystals (XNCs) with a uniform size can be produced by a mild ultrasonic treatment introduced to the suspensions of xylan crystals. Noncrystalline and soluble xylans stabilize oil-in-water emulsions by forming a gel network, which is viscosity dependent. XNCs have better oil-in-water emulsifying properties than noncrystalline and soluble xylans, since XNCs can be adsorbed onto oil–water interfaces at a high density to form stable Pickering emulsions. XNCs demonstrate comparable emulsifying properties compared to synthetic emulsifiers, e.g., T80 and TX100. Considering that the production of XNCs is completely green without any acid treatments or chemical derivatizations, these results suggest that XNCs have great potential to substitute synthetic chemicals in emulsification.

## Figures and Tables

**Table 1:** Xylans separated through different extraction methods.

Xylan Fractions	Extraction Methods	Yield %
X1	1 wt% NaOH	10.77 ± 0.21
X2	2 wt% NaOH	21.55 ± 0.10
X3	4 wt% NaOH (successive after 2 wt%)	7.71 ± 0.04
X4	6 wt% NaOH (successive after 2 and 4 wt%)	9.08 ± 0.12

Note: the yields of xylans corresponded only to the indicated extraction step for successive extraction and were calculated based on the starting holocellulose.

**Table 2:** Chemical compositions of the xylan separated through different extraction methods.

Samples	Arabinose %	Galactose %	Glucose %	Xylose %	Uronic Acid %	Lignin%
X1	12.72 ± 0.06	2.04 ± 0.07	5.59 ± 0.05	51.29 ± 0.36	6.77 ± 0.14	7.60 ± 1.55
X2	10.99 ± 0.38	2.18 ± 0.00	7.06 ± 0.12	67.12 ± 1.01	2.77 ± 0.08	1.54 ± 0.11
X3	6.66 ± 0.03	0.10 ± 0.08	6.06 ± 0.01	81.42 ± 0.64	2.24 ± 0.01	1.19 ± 0.51
X4	1.63 ± 0.16	0.27 ± 0.68	6.84 ± 2.79	74.56 ± 0.94	1.50 ± 0.01	0.88 ± 0.05

**Table 3:** Molecular structures of separated xylan fractions.

Samples	Mn	Mw	Polydispersity Index	* Degree of Substitution
X1	54,400	85,100	1.56	0.38
X2	52,800	95,400	1.81	0.21
X3	45,900	75,200	1.64	0.11
X4	31,300	51,400	1.64	0.04

* Calculated by the content ratios of (arabinose + uronic acid)/xylose.

**Table 4:** Comparisons with other natural polysaccharide nanocrystals in the Pickering emulsion.

Emulsifier	Oil–Water Ratio	Stability Condition	* ECI (%)	Reference
CNC	1:4 (*w*:*w*)	Measured right after	≥55	[17]
CNC with CNF	1:99 (*w*:*w*)	Stand for 7 days	95–0	[15]
CNC from bacterial cellulose	3:7	Centrifuged at 4000 g for 10 min	80–10	[33]
CNC	3:7	Centrifuged at 4000 g for 10 min	≥70	[34]
Starch nanocrystals	1:1 (*v*:*v*)	Stand for 2 months	20–0	[21]
Starch nanocrystals	1:3 to 3:1	Stand for 24 h	84–10	[35]
Chitin nanocrystals	2:8	Measured right after	70–30	[16]
Chitin nanocrystals	2:8	Measured right after	60–10	[19]
XNC	2:8 (*w*:*w*)	Centrifuged at 4000 g for 10 min/stand for 7 days	Close to 0	This work

* Appropriate estimation according to the pictures or tables in the literature.

## Supplementary Materials

The following are available online at https://www.mdpi.com/article/10.3390/nano11102558/s1, Figure S1: Polarizing microscope images of xylan components in 0.5 wt% aqueous solution, Figure S2: Dispersibility/solubility of xylans with different crystallinity, Figure S3: The emulsion cream index of X1-X4 after standing for seven days, Figure S4: Pictures of emulsion after standing for 7 days at different emulsifier concentration.
