# Supplementary material for: Highly Stable Pickering Emulsions with Xylan Hydrate Nanocrystals
Source: Nanomaterials (Basel). 2021 Sep 29;11(10):2558. doi: 10.3390/nano11102558 (PMC8537821; doi:10.3390/nano11102558)
Supplement: Supplementary file 1 [file nanomaterials-11-02558-s001.zip › nanomaterials-1381152-supplementary.pdf]

Supplementary Materials

Highly Stable Pickering Emulsions with Xylan Hydrate Nano-Crystals

Shanyong Wang and Zhouyang Xiang *

State Key Laboratory of Pulp and Paper Engineering, South China University of Technology, Guangzhou 510640, China; feshanyong@mail.scut.edu.cn
* Correspondence: fezyxiang@scut.edu.cn; Tel.: +86-20-87113753

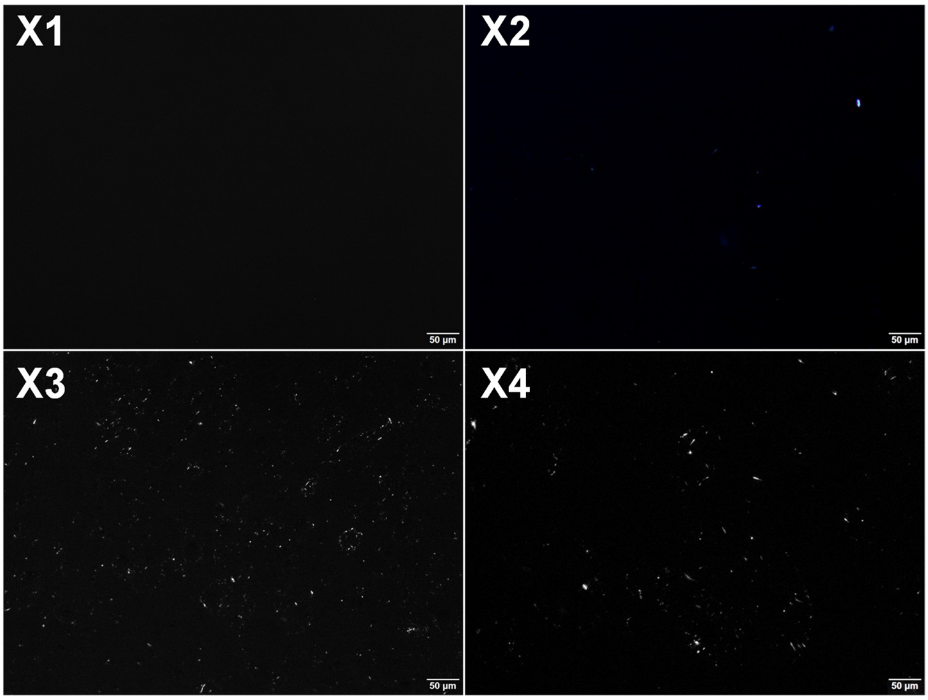

Figure S1. Polarizing microscope images of xylan components in 0.5 wt% aqueous solution.

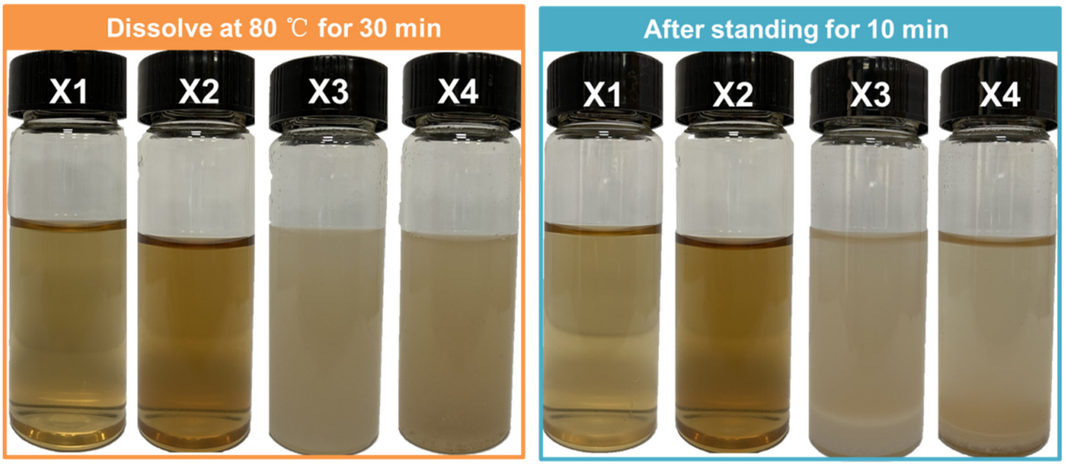

Figure S2. Dispersibility/solubility of xylans with different crystallinity.

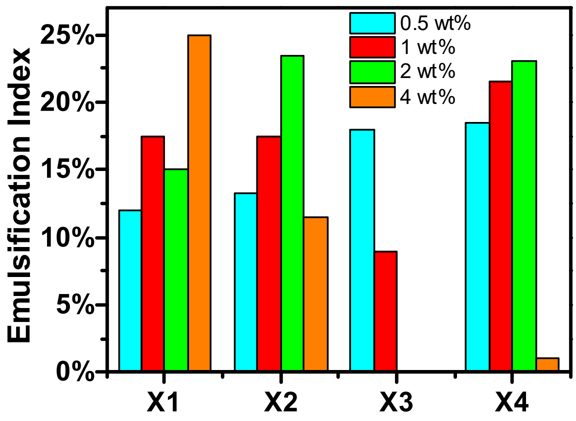

Figure S3. The emulsion cream index of X1-X4 after standing for seven days.

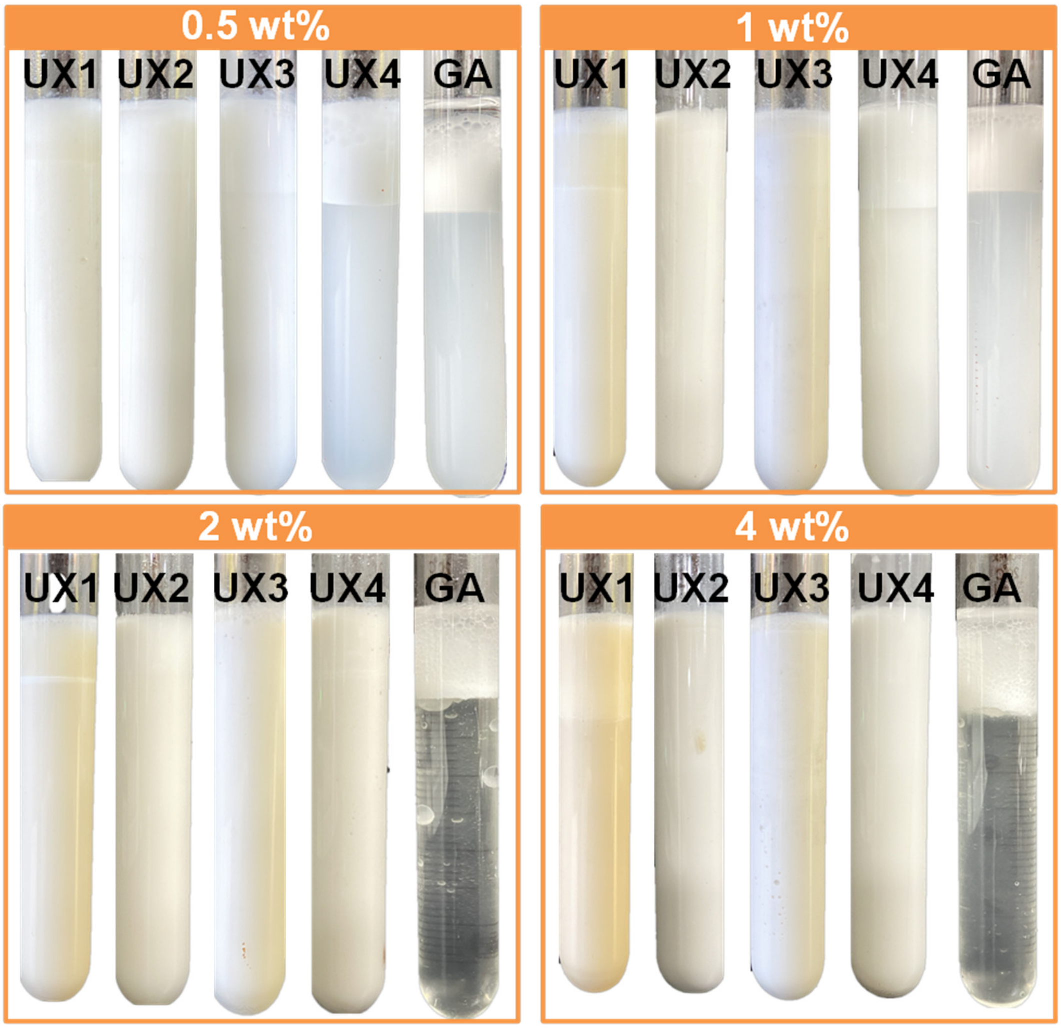

Figure S4. Pictures of emulsion after standing for 7 days at different emulsifier concentration.
